# The impact of exposure to HPV related information and injunctive norms on young women's intentions to receive the HPV vaccine in China: A structural equation model based on KAP theory

**DOI:** 10.3389/fpubh.2022.1102590

**Published:** 2023-01-16

**Authors:** Yi Wang, Yubing Chen, Sheng Bao

**Affiliations:** School of Journalism and Communication, Huaqiao University, Xiamen, China

**Keywords:** intention to receive the HPV vaccine, Chinese young women, HPV related information exposure, injunctive norms, structural equation model, KAP theory

## Abstract

**Background:**

The HPV vaccination is a crucial line of defensing against cervical cancer. As a result of government support and positive publicity from the majority of media, a craze for HPV vaccination has occurred in China. Besides, the intentions to get the HPV vaccine among women of appropriate age is also influenced by families' and friends' attitudes and perceptions toward HPV vaccine. Therefore, the purpose of this study was to investigate how HPV related information exposure and injunctive norms affect young Chinese women's intentions to receive the HPV vaccine.

**Methods:**

A structural equation model was developed based on KAP theory, and 567 effective questionnaires were collected through an online survey. We used SPSS 26.0 for the reliability and validity analysis and the differential testing of demographic characteristics, and Amos 26.0 for the goodness-of-fit analysis and paths testing of the model.

**Results:**

Our findings showed that (1) intention to receive HPV vaccine differed significantly in age (*P* = 0.046), educational background (*P* = 0.001), and occupation (*P* = 0.004). (2) Exposure to HPV related information positively affected knowledge about HPV (β = 0.316, *P* < 0.001) and intention to receive HPV vaccine (β = 0.141, *P* < 0.001). (3) Knowledge about HPV positively affected attitude toward HPV vaccine (β=0.341, *P* < 0.001), but negatively affected intention to receive HPV vaccine (β = −0.148, *P* < 0.05), and attitude toward HPV vaccine positively affected intention to receive HPV vaccine (β = 0.594, *P* < 0.001). (4) Injunctive norms positively affected attitude toward HPV vaccine (β = 0.362, *P* < 0.001) and intention to receive HPV vaccine (β = 0.420, *P* < 0.001).

**Conclusions:**

Exposure to HPV related information influenced young Chinese women's intentions to receive the HPV vaccine and related knowledge, that is, the more frequently they were exposed to HPV related information, the stronger their intentions to receive the vaccine and the higher their HPV knowledge. Also, the perception and support of HPV vaccination by people around them will further influence their attitudes and intentions to receive the HPV vaccine.

## 1. Introduction

HPV, known as human papilloma virus, is the most common sexually transmitted virus worldwide. HPV infection is usually unnoticed, and persistent infection with high-risk HPV types in particular is the leading cause of diseases such as penile cancer, anal cancer, precancerous cervical lesions and cervical cancer (1, 2). According to the statistics, 604,000 new cases of cervical cancer and 342,000 deaths occurred globally in 2020 (3). More than 85% of these cervical cancer cases and deaths occurred in developing countries, and as the world's largest developing country, China has 110,000 new cases and a high mortality rate of 55% per year (4). To prevent various health problems caused by HPV infection, HPV vaccination has become one of the most important countermeasures (5). In recent years, the Chinese government has been actively promoting the scientific publicity and pilot work of HPV vaccination, such as facilitating Guangdong, Hainan and Fujian provinces to successively launch the implementation of free and voluntary domestic bivalent HPV vaccination for girls of appropriate age in the whole province, while some cities have provided them flat-rate subsidies for HPV vaccination.

However, awareness of HPV and the vaccine remains at a relatively low level. A meta-analysis showed that the pooled awareness and knowledge rates about HPV vaccination were 15.95 % and 17.55 % respectively (6). And the current HPV vaccine coverage in China was low, with <3% for the adolescent population and <6% for the entire population (7).^1^ This number is significantly lower than the global HPV vaccine coverage (15%) announced by the World Health Organization for 2020 (8).

In the era of social media, the vast amount of information sources related to HPV vaccine provides more and more channels for the public to know about HPV, which may implicitly influence the public's attitudes and beliefs, and then may influence their behaviors (9). And the behavioral decisions of the public are also influenced by factors in the social environment including their perceived social norms. Numerous studies in recent years have emphasized that vaccination decisions should be comprehended in a broader sociocultural context and, in particular, should take the effects of social norms into account (10, 11). China's unique cultural context also provides special conditions for examining the relationship between social norms and intention to receive HPV vaccine. Hofstede noted that China scores higher on the values of collectivism than many western countries (12). Chinese society has a strong collectivistic culture atmosphere, and compared with an individualistic culture, people raised in a collectivistic culture show more conformity to injunctive norms. Therefore, this study focuses on the influential mechanism of injunctive norms on the intentions to receive HPV vaccine among Chinese women of appropriate age under the special cultural context of China.

Given the above background, the aim of this study was to investigate how exposure to HPV related information and injunctive norms affect the intentions to receive HPV vaccine among the young women in China. The Knowledge-Attitude-Practice Theory, a classical theory in the field of health communication, was also adopted to construct a structural equation model in an attempt to verify the influence paths of information exposure and injunctive norms on HPV vaccination intention among the Chinese women of appropriate age.

## 2. Literature review and hypotheses

### 2.1. Exposure to HPV related information

Previous studies into health information exposure mainly include three aspects: first, studies on the source types of health information exposure. In the Internet era, people's access to information is diversified and complicated, and information from different channels has different impacts on people's attitudes and behaviors (13–15). Second, studies on the types of health information exposure behaviors. Lambert et al. and Wilson classified health information behavior (HIB) into three types: active HIB (conscious and goal-oriented seeking of information), passive HIB (someone encounters a certain type of information without consciously searching for it), and avoidance HIB (the conscious decision not to search for and encounter a certain type of information) (16–19). Different patterns of information exposure also have different effects on individual behavior (20). Third, studies on the impacts of health information exposure on health behaviors. A series of observational studies found that frequent exposure to health information was associated with healthier behaviors, such as regular physical exercise, fruit and vegetable intake, and smoking and alcohol quitting (21).

Existing studies have explored the relationship between exposure to HPV information and intention to receive HPV vaccine in many countries. For example, in a study of parents' intention to vaccinate their children against HPV, McRee et al. (22) found that parents' access to HPV related health information through the Internet was positively associated with their intention to vaccinate their children, and repeated exposure to controversial information about the HPV vaccine would reduce the public support for the vaccine. Moreover, several lines of evidence also suggested that HPV information exposure is closely related to the promotion of related health knowledge. Ortiz and Lyson have pointed out that exposure to HPV related social media content is associated with increased HPV awareness and knowledge, and the use of social media for health interventions is considered an effective means to increase HPV knowledge among adolescents (23, 24). Li et al. (25) also found that participants' frequency of using professional sources to search for information could influence their level of knowledge about HPV and HPV vaccine. Therefore, we made the following hypotheses:

*H1: Exposure to HPV related information positively affects the knowledge level about HPV among young women*.
*H2: Exposure to HPV related information positively affects the intention to receive the HPV vaccine among young women*.

### 2.2. Knowledge-attitude-practice theory

Knowledge-attitude-practice theory (KAP Theory) is used to explain the relationships between the three levels of health behavior change (26). And it has become a research tool for developing public health strategies by exploring the knowledge, attitudes, and health behaviors of populations with health problems. This theory emphasizes the key role of knowledge and attitude in behavioral decision making, explains the generation and change of health behaviors, and in turn provides important guidance for the implementation of health education and behavioral interventions. Since it was proposed, this theory has been widely applied and verified in various fields, including infectious diseases such as AIDS (27) and hepatitis B (28), cancers such as breast cancer (28, 29), chronic diseases such as diabetes (30) and hypertension (31), food control (32, 33) and diet nutrition (34, 35), etc.

On the topic of vaccination, Chen et al. (36) investigated influenza vaccination among healthcare workers in Chongqing and noted that influenza-related knowledge was positively associated with attitude and vaccination practice, with a strong mediating effect of attitude between knowledge and practice. In a survey on the current knowledge, attitude and practice of HPV vaccination among female college students in Hong Kong, Leung and Law (37) found that female college students with high HPV vaccine knowledge and positive attitude toward HPV vaccine were more likely to receive the HPV vaccine. Additionally, individuals with high levels of knowledge are more likely to recognize the efficacy of vaccines and thus have a positive attitude toward HPV vaccination. Likewise, a systematic review pointed out that immigrant parents with generally low knowledge of HPV related diseases and vaccines tend to have a negative attitude toward vaccinating their children against HPV (38). Hence, we made the following hypotheses:

*H3: Knowledge about HPV positively affects the intention to receive HPV vaccine among young women*.
*H4: Knowledge about HPV positively affects the attitude toward HPV vaccine among young women*.
*H5: Attitude toward HPV vaccine positively affects the intention to receive HPV vaccine among young women*.

### 2.3. Injunctive norms

Injunctive norms are one kind of social norms that refers to the perception of social approval for a certain behavior, which reflects the pressure of individuals to comply with it (39). If the majority approves or supports a behavior, the likelihood of individuals performing the same behavior increases. The effect mechanism of injunctive norms on behavior is based on the external social sanctions and incentives. Behavior that is consistent with the majority's views can obtain the expected spiritual or material benefits from the group. The injunctive norms essentially reflect the mainstream values of society, that is, the value judgment tendency of the majority (40).

Current studies have found that injunctive norms have a significant role in advising behaviors such as smoking cessation, alcohol cessation, and healthy eating (41–44). Likewise, several studies demonstrated that injunctive norms also had an effect on individuals' intention to receive the HPV vaccine. Using a comparison of HPV vaccination intentions among young women in China and the United States, Pan et al. (45) concluded that contradictory information and injunctive norms from social media positively affected attitudes and intentions toward HPV vaccination. Meanwhile, it has also been demonstrated that parents' intentions to vaccinate adolescents against HPV are significantly associated with the perceived injunctive norms (46). Hence, we made the following hypotheses:

*H6: Injunctive norms positively affect the intention to receive the HPV vaccine among young women*.
*H7: Injunctive norms positively affect the attitude toward HPV vaccine among young women*.

**Figure 1:**
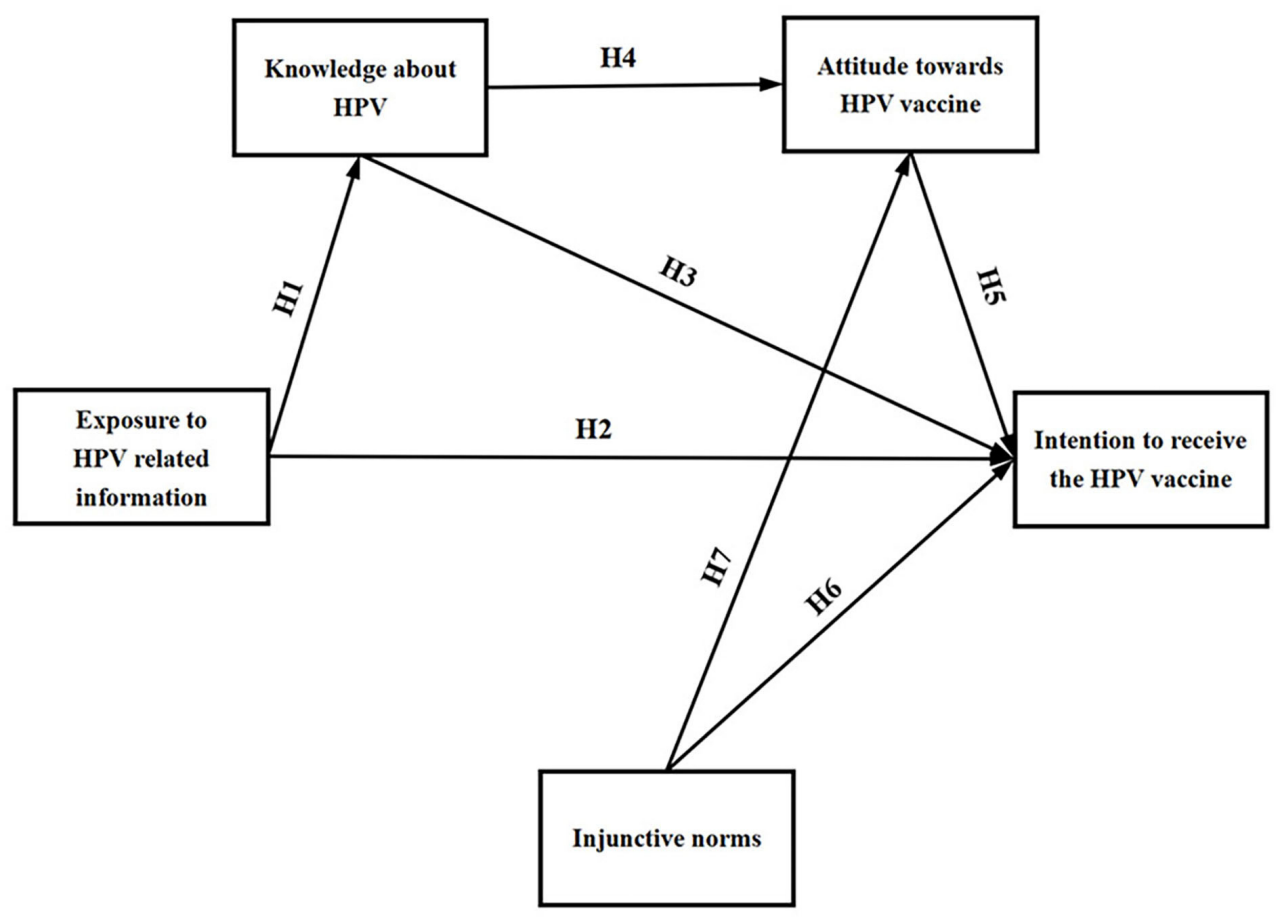
Research model.

The research model is shown in Figure 1, which contains five main variables and seven hypotheses.

## 3. Materials and methods

### 3.1. Participants

The World Health Organization classifies young people as being aged 15–44 years (47), an age group similar to the age at which the HPV vaccine is indicated (women aged 9–45 years) (48); therefore, participants in this study were required to be women aged 15–44 years who had not received the HPV vaccine. Additionally, this study focused on the impact of HPV related information exposure on vaccination intentions, participants who are unaware of the HPV vaccine will be excluded from the final sample by the question “Have you heard of the HPV vaccine?” in the questionnaire.

### 3.2. Data collection

This study adopted a random online questionnaire method, relying on Wenjuanxing (www.wjx.cn), an online questionnaire platform in China, to design the questionnaire and deliver it to the target population through social media such as WeChat and QQ. The privacy of questionnaire participants will be strictly protected. Data collection was carried out in two stages:

The first stage was a pre-survey. Before the formal survey, the preliminary analysis of the reliability and validity of the questionnaire was conducted. In this stage, a total of 136 questionnaires were collected and 78 were effectively completed, and the questionnaire items were adjusted and modified according to the analysis results.

The second stage was the formal survey. This stage started on October 16, 2022 and ended on October 31, 2022, with a total of 1,169 questionnaires collected. The second stage was the formal survey. This stage started on October 16, 2022 and ended on October 31, 2022, with a total of 1,169 questionnaires collected. After eliminating 602 invalid questionnaires with <60 s of response time, incomplete answers, too many extreme answers, contradictory answers, and completed by participants who did not meet the requirements of this study, the final number of effective questionnaires obtained was 567, and the effective rate was 48.5%.

The demographic characteristics of the participants are shown in Table 1. In this study, the demographic information of the participants mainly included their age, educational background, profession, occupation, and monthly income. The 567 valid participants were mainly distributed in the 20–24 age group (49.6%), followed by the 15–19 age group (27%) and the 25–29 age group (14.5%). The majority of participants held a bachelor's degree or above, with 62.1% holding a bachelor's degree and 20.3% holding a master's degree or above. Only 17 participants (3%) had a medical background. As for occupation, the majority were students (65.6%), followed by enterprise employees (12.7%). Since most of the participants were students, 66.3% of them had a monthly income of ≤3,000 yuan, followed by 3,001–6,000 yuan (18.7%). On the whole, the samples met the characteristics requirements of the target group in our study.

**Table 1:** Demographic characteristics of the participants (*N* = 567).

**Characteristic**	**Demographic information**	**Frequency**	**%**
Age	15–19	153	27
	20–24	281	49.6
	25–29	82	14.5
	30–34	24	4.2
	35–39	18	3.2
	40–44	9	1.6
Education background	Junior college or lower	100	17.6
	Bachelor's degree	352	62.1
	Master's degree or higher	115	20.3
Major	Medical	17	3
	Non-medical	550	97
Occupation	Students	372	65.6
	Civil servants or public institution personnel	44	7.8
	Employees of enterprises	72	12.7
	Self-employed or private owners	25	4.4
	Medical practitioners	4	0.7
	others	50	8.8
Monthly income (CNY)	≤3,000	376	66.3
	3,001–6,000	106	18.7
	6,001–9,000	46	8.1
	9,001–12,000	17	3
	≥12,001	22	3.9

CNY, China Yuan.

### 3.3. Measures

#### 3.3.1. Exposure to HPV related information

The measure of exposure to HPV related information was adapted from Mars et al. (49). The scale measures information exposure in both active and passive ways, and consists of three items: “Have you ever come across and viewed HPV-related information on Weibo, WeChat, Zhihu, Douban or other online media?”, “Have you ever looked for HPV-related information using the search function of WeChat or other search engines?” and “Have you ever discussed HPV with others through WeChat, QQ or other channels?”. These items use five-point Likert scales ranging from 1 “never” to 5 “always”. And the Cronbach's Alpha was good, >0.8 (α = 0.826, M = 3.434, SD = 0.850).

#### 3.3.2. Knowledge about HPV

Knowledge about HPV was measured by eight items, adapted from Waller et al. (50). The original scale contains three parts: HPV knowledge, HPV examination and HPV vaccine. In this study, 4 items were selected in each part of HPV knowledge and HPV vaccine, a total of 8 items were translated into Chinese. Participants were asked about their opinions on the following 8 items in the questionnaire: “There are many types of HPV,” “HPV can cause cervical cancer,” “HPV can be passed on during sexual intercourse,” “Men can also get HPV,” “The HPV vaccines offer protection against most cervical cancers,” “Girls who have had the HPV vaccine still need a regular (Pap test/Smear test/Pap smear test) when they are older,” “The HPV vaccines are most effective if given to people who have never had sex,” and “The higher the valent of the HPV vaccine, the more types of HPV it can prevent.” These items use five-point Likert scales ranging from 1 “strongly disagree” to 5 “strongly agree.” The Cronbach's Alpha was much higher than the original scale (α = 0.904, M = 4.173, SD = 0.645).

#### 3.3.3. Attitude toward HPV vaccine

The measure of attitude toward HPV vaccine was adapted from Askelson et al. (51) and consisted of five items: “Vaccinating is necessary,” “Vaccinating is a good idea,” “Vaccinating is beneficial,” “Vaccinating is safe” and “Vaccinating is important.” These items use five-point Likert scales ranging from 1 “strongly disagree” to 5 “strongly agree.” The Cronbach's Alpha was excellent (α = 0.961, M = 4.428, SD = 0.682).

#### 3.3.4. Injunctive norms

Injunctive norms mainly refer to the attitudes and perceptions of people around you regarding HPV vaccination. And this variable was measured by four items, adapted from Lee and Su (52): “How likely it is that your family members would feel about you get the HPV vaccine?”, “How likely it is that your close friends would feel about you get the HPV vaccine?”, “How likely it is that your classmates or colleagues would feel about you get the HPV vaccine?” and “How likely it is that people of your age would feel about you get the HPV vaccine?”. These items use five-point Likert scales ranging from 1 “strongly disapprove” to 5 “strongly approve”. And the Cronbach's Alpha was excellent (α = 0.900, M = 4.312, SD = 0.691).

#### 3.3.5. Intention to receive the HPV vaccine

Intention to receive the HPV vaccine was measured by three items, adapted from Nan and Madden (53) and Fazekas et al. (54): “How likely would you be to get the HPV vaccine sometime soon?”, “How likely would you be to actually get the HPV vaccine once it is available?” and “How likely would you be to get the HPV vaccine if it is provided for free in the next three years?”. These items use five-point Likert scales ranging from 1 “very unlikely” to 5 “very likely”. The Cronbach's Alpha was acceptable (α = 0.767, M = 4.316, SD = 0.739).

### 3.4. Data analysis methods

In this study, IMB-SPSS 26.0 and IBM-SPSS-Amos 26.0 were used for data processing and analysis. We used SPSS for descriptive statistical analysis, reliability analysis and one-way analysis of variance (one-way ANOVA). Amos was used for confirmatory factor analysis (CFA), goodness-of-fit testing and path testing of the structural equation model.

## 4. Data analysis results

### 4.1. Differential testing of demographic characteristics

To examine differences in intention to receive the HPV vaccine among demographic characteristics, we performed a one-way ANOVA using SPSS. The precondition of one-way ANOVA is to ensure the homogeneity of variance of each independent group, so the homogeneity of variance should be tested first. In the homogeneity of variance testing results, if the *P* > 0.05, the result of ANOVA is taken as the final result; if the *P* < 0.05, the variance is not homogeneous (55). According to Liu's suggestion (56), Welch ANOVA can be applied in this case when the variance is not homogeneous. Therefore, this study used Welch ANOVA to perform differential test differences among groups with heterogeneous variances. Table 2 shows the results of ANOVA. The variances of education background and occupation are not homogeneous, so Welch method was adopted for testing. The results showed that the three control variables (age, educational background and occupation) had significant differences in HPV vaccination intention (*P* < 0.05). Specifically, people aged 20–24, those with a master's degree or above, medical practitioners, civil servants and those working in public institutions were more likely to get HPV vaccine.

**Table 2:** Differential testing of demographic characteristics in intention to receive the HPV vaccine (*N* = 567).

**Characteristic**	**Demographic information**	**Mean**	**SD**	***P***
Age	15–19	4.1765	0.734	0.046
	20–24	4.4033	0.673	
	25–29	4.3252	0.851	
	30–34	4.2500	0.744	
	35–39	4.3333	0.915	
	40–44	4.0000	1.014	
Education background^*^	Junior college or lower	4.0500	0.845	0.001
	Bachelor's degree	4.3561	0.712	
	Master's degree or higher	4.4232	0.669	
Major	Medical	4.2353	0.984	0.649
	Non-medical	4.3182	0.731	
Professional^*^	Students	4.2751	0.722	0.005
	Civil servants or public institution personnel	4.6515	0.639	
	Employees of enterprises	4.2917	0.859	
	Self-employed or private owners	4.0000	0.977	
	Medical practitioners	4.6667	0.471	
	Other	4.4867	0.491	
Monthly income (CNY)	≤3,000	4.2580	0.740	0.067
	3,001–6,000	4.4277	0.635	
	6,001–9,000	4.5217	0.635	
	9,001–12,000	4.2353	1.177	
	≥12,001	4.3939	0.877	

Variables with “^*^” indicate the heterogeneity of variance, using the Welch ANOVA.

SD, standard deviation.

### 4.2. Confirmatory factor analysis

**Figure 2:**
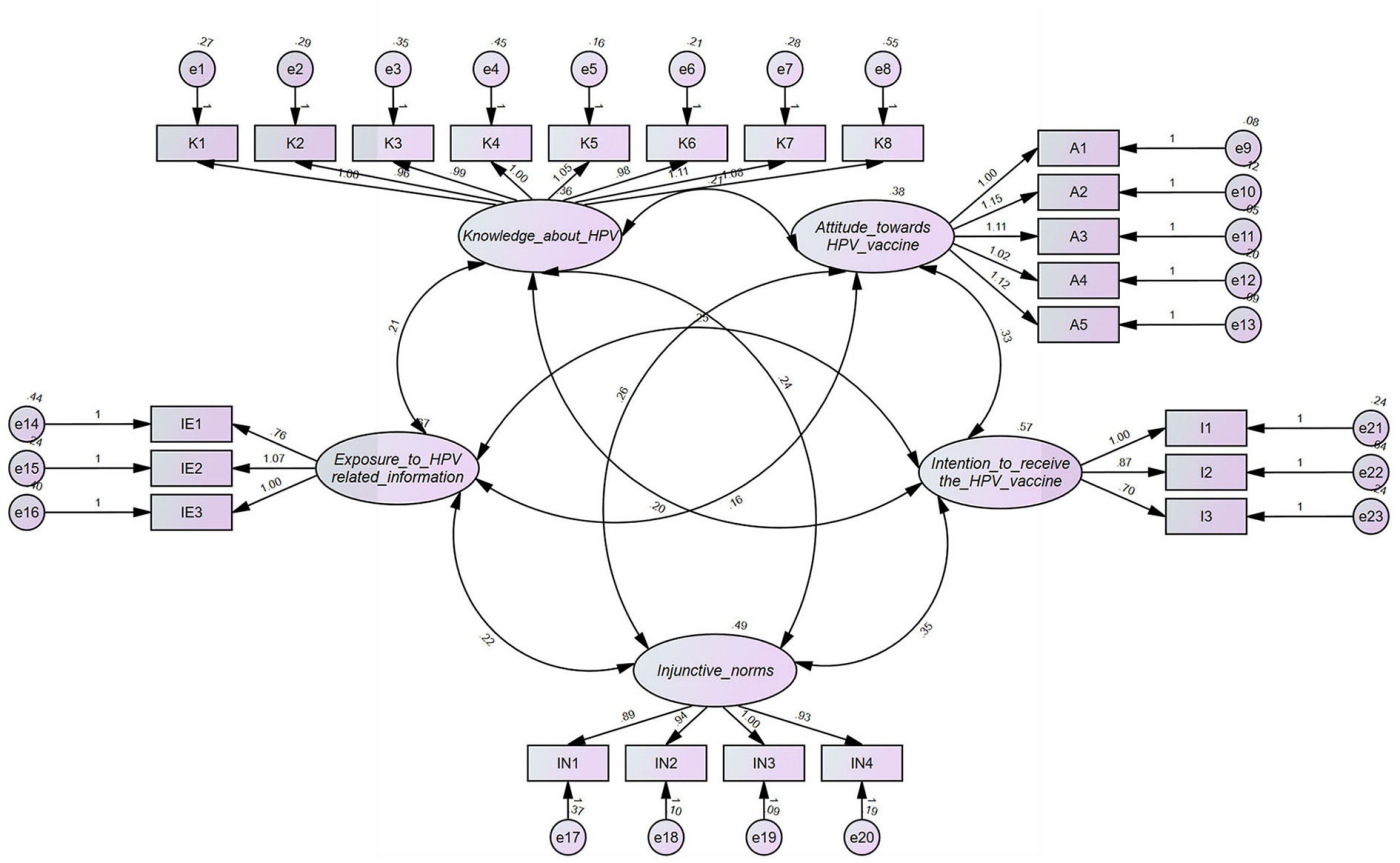
Confirmatory factor analysis.

In order to measure the variables in the model, we referred to the maturity scale of previous studies, and adapted it to fit within the scope of this study. And its validity was tested by confirmatory factor analysis (CFA) method (see Figure 2). Confirmatory factor analysis is the process of using sample data to verify whether the structural models (the corresponding relationships between scale items and latent variables, the relationships between latent variables) which has been made according to some theories and prior knowledge are consistent with the actual data. This method was operated in Amos, and by running all the latent variables in the software, the factor loadings for each item were obtained. As shown in Table 3, the factor loadings of each item are from 0.631 to 0.95, all of which are >0.6, indicating that the measures of each latent variable have high correlation and high convergent validity, and the items designed in the questionnaire are reasonable.

**Table 3:** Factor loadings.

**Latent Variables**		**Items**	**Factor loadings**
Exposure to HPV related information	IE1	Have you ever come across and viewed HPV-related information on Weibo, WeChat, Zhihu, Douban or other online media?	0.688
	IE2	Have you ever looked for HPV-related information using the search function of WeChat or other search engines?	0.873
	IE3	Have you ever discussed HPV with others through WeChat, QQ or other channels?	0.793
Knowledge about HPV	K1	There are many types of HPV	0.754
	K2	HPV can cause cervical cancer	0.731
	K3	HPV can be passed on during sexual intercourse	0.705
	K4	Men can also get HPV	0.667
	K5	The HPV vaccines offer protection against most cervical cancers	0.841
	K6	Girls who have had the HPV vaccine still need a regular (Pap test/Smear test/Pap smear test) when they are older	0.783
	K7	The HPV vaccines are most effective if given to people who have never had sex	0.781
	K8	The higher the valent of the HPV vaccine, the more types of HPV it can prevent	0.660
Attitude toward HPV vaccine	A1	Vaccinating is necessary	0.909
	A2	Vaccinating is a good idea	0.900
	A3	Vaccinating is beneficial	0.950
	A4	Vaccinating is safe	0.815
	A5	Vaccinating is important	0.921
Injunctive norms	IN1	How likely it is that your family members would feel about you get the HPV vaccine?	0.717
	IN2	How likely it is that your close friends would feel about you get the HPV vaccine?	0.897
	IN3	How likely it is that your classmates or colleagues would feel about you get the HPV vaccine?	0.915
	IN4	How likely it is that people of your age would feel about you get the HPV vaccine?	0.833
Intention to receive the HPV vaccine	I1	How likely would you be to get the HPV vaccine sometime soon?	0.839
	I2	How likely would you be to actually get the HPV vaccine once it is available?	0.631
	I3	How likely would you be to get the HPV vaccine if it is provided for free in the next 3 years?	0.732

### 4.3. Reliability and validity testing

In this study, Cronbach's Alpha was used as the evaluation criterion of reliability. Fornell and Larcker (57) point out that when the α is >0.7, the internal reliability of the questionnaire items is considered acceptable. The Cronbach's Alpha values for each variable in Table 4 are between 0.767 and 0.961, all <0.7, suggesting good reliability and strong internal consistency of the measurement items in this study.

**Table 4:** Reliability and convergence validity testing results.

**Latent variables**	**Cronbach's Alpha**	**CR**	**AVE**
Intention to receive the HPV vaccine	0.767	0.781	0.546
Exposure to HPV related information	0.826	0.830	0.621
Injunctive norms	0.900	0.907	0.711
Attitude toward HPV vaccine	0.961	0.955	0.810
Knowledge about HPV	0.904	0.907	0.551

CR, composite reliability; AVE, average variance extracted.

The validity testing includes content validity, convergent validity and discriminant validity. Firstly, this study was based on validated scales and adequate pre-research, so the content validity was well guaranteed. Secondly, according to the results in Tables 3, 4, the factor loadings of each item was greater than the recommended value of 0.6 (58), the composite reliability (CR) of each latent variable was greater than the recommended value of 0.7 (57), and the average variance extraction value (AVE) was greater than the recommended value of 0.5 (59). All the three criteria of convergent validity were satisfied, indicating that all the measured items had good convergent validity. The discriminant validity is evaluated by the comparison between the square root of AVE and the correlation coefficient between latent variables. As shown in Table 5, the square root of AVE (bolded values) of all latent variables involved in this study was greater than the correlation coefficient between this latent variable and other latent variables, which suggested that the measurement items in this study were of good discriminant validity.

**Table 5:** Discriminant validity testing results.

**Latent variables**	**Intention to receive the HPV vaccine**	**Exposure to HPV related information**	**Injunctive norms**	**Attitude toward HPV vaccine**	**Knowledge about HPV**
Intention to receive the HPV vaccine	**0.739**				
Exposure to HPV related information	0.249	**0.788**			
Injunctive norms	0.354	0.217	**0.843**		
Attitude toward HPV vaccine	0.326	0.16	0.259	**0.900**	
Knowledge about HPV	0.202	0.213	0.237	0.208	**0.742**

The bold values indicate the square root of AVE.

Through the above analysis, it can be seen that the questionnaire design of this study was reasonable and had good reliability and validity. To measure the valid and proper design of the structural equation model, further model fitting analysis can be conducted.

### 4.4. Model fitting

In this study, the structural equation model was constructed and evaluated using Amos. And the following suggested criteria from Hooper et al. (60) were adopted for the model fit: (1) The chi-square to degree of freedom ratio (x^2^/df), should be between 1 and 5; (2) The root means square error of approximation (RMSEA), should be < 0.08; (3) The standardized root means square residual (SRMR), should be <0.08; (4) Tucker-Lewis index (TLI), should be >0.95; (5) The normed fit index (NFI), should be >0.9; (6) Comparative fit index (CFI), should be >0.9.

The initial model fit was evaluated with the following results: x^2^/df = 3.504, RMSEA = 0.067, SRMR = 0.097, TFI = 0.933, NFI = 0.920, CFI = 0.941. Two indexes, SRMR and TFI, did not meet the suggested criteria, so the initial model was less than ideal and needed to be modified. According to the Modification index (MI) in the Amos output, covariant relationships were established to reduce the chi-square value and further fit the model. The results of the model fit after modification are shown in Table 6: *x*^2^/df = 2.782, RMSEA = 0.056, SRMR = 0.039, TFI = 0.952, NFI = 0.937, and CFI = 0.958. All the indexes after modification met the fit criteria, and the model is good in fitting effect and well adapted to the sample data.

**Table 6:** Model fitting indexes after modification.

**Index**	**x^2^/df**	**RMSEA**	**SRMR**	**TLI**	**NFI**	**CFI**
Observed value	2.782	0.056	0.039	0.952	0.937	0.958
Ideal value	<5	<0.08	<0.08	>0.95	>0.9	>0.9

RMSEA, root means square error of approximation; SRMR, standardized root means square residual; TLI, Tucker-Lewis index; NFI, normed fit index; CFI, comparative fit index.

### 4.5. Hypothesis testing

**Figure 3:**
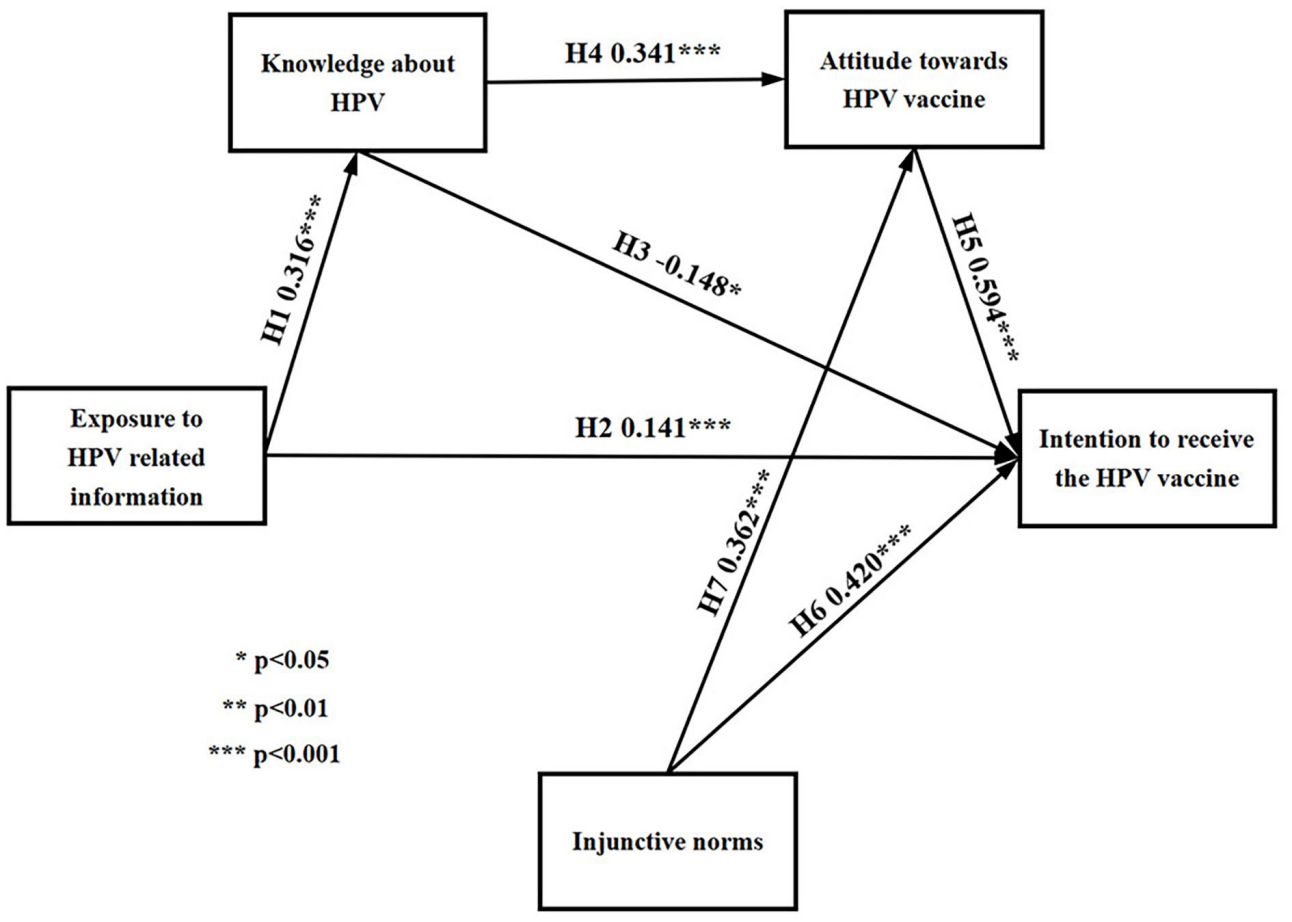
Path coefficients of the proposed model. *p < 0.05, **p < 0.01, ***p < 0.001.

There are seven paths in this study. Amos was used to analyze the paths, and then the research hypotheses were tested. The final path coefficient of proposed model and hypotheses testing results are presented in Figure 3, Table 7. The results showed that all the seven paths were at the significant level of *P* < 0.05, indicating that the connectivity of the paths could be supported. Exposure HPV related information positively affected knowledge about HPV (β = 0.316, *P* < 0.001) and intention to receive the HPV vaccine (β = 0.141, *P* < 0.001), so H1 and H2 were supported. Knowledge about HPV positively affected attitude toward HPV vaccine (β = 0.341, *P* < 0.001), but negatively affected intention to receive the HPV vaccine (β = −0.148, *P* < 0.05). H4 was supported, but H3 was not. Attitude toward HPV vaccine positively affected intention to receive the HPV vaccine (β = 0.594, *P* < 0.001), and H5 was supported. Injunctive norms positively affected attitude toward HPV vaccine (β = 0.362, *P* < 0.001) and intention to receive the HPV vaccine (β = 0.420, *P* < 0.001), H6 and H7 were supported.

**Table 7:** Hypothesis testing results.

**Hypotheses**	**Paths**	**Path coefficients (β)**	***T*-statistics**	***P***	**Results**
H1	Exposure to HPV related information → Knowledge about HPV	0.316	8.783	<0.001	Supported
H2	Exposure to HPV related information → Intention to receive the HPV vaccine	0.141	3.572	<0.001	Supported
H3	Knowledge about HPV → Intention to receive the HPV vaccine	−0.148	−2.335	0.020	Not supported
H4	Knowledge about HPV → Attitude toward HPV vaccine	0.341	7.22	<0.001	Supported
H5	Attitude toward HPV vaccine → Intention to receive the HPV vaccine	0.594	9.826	<0.001	Supported
H6	Injunctive norms → Intention to receive the HPV vaccine	0.420	7.644	<0.001	Supported
H7	Injunctive norms → Attitude toward HPV vaccine	0.362	9.142	<0.001	Supported

## 5. Discussion

### 5.1. The effect of exposure to HPV related information on knowledge about HPV and intention to receive the HPV vaccine

In recent years, the incidence and mortality rate of cervical cancer in China has been on the rise, posing a serious threat to the lives of Chinese women. And HPV vaccination, as a primary prevention measure, is the most crucial line of defense against cervical cancer. This study aimed to investigate how exposure to HPV related information and injunctive norms affect young Chinese women's intention to receive the HPV vaccine. Foremost, our results provided evidence to positive effect of HPV related information exposure on the knowledge about HPV, which is in line with previous studies (61). Furthermore, we also found that exposure to HPV related information positively affected intention to receive HPV vaccine. This finding echoes the existing studies related to HPV vaccines, as demonstrated in the research by Chen et al. (62), which confirmed that people with more knowledge about HPV vaccines had more positive attitudes and similarly had stronger behavioral intentions to get vaccinated. In another cross-sectional study of intention to receive COVID-19 vaccine, it was also concluded that exposure to information about COVID-19 vaccine was a significant predictor of vaccination intention (63).

Information exposure plays a prominent role in determining the HPV-related knowledge level and intention to vaccinate among the public. This study verified the applicability of the above two findings in mainland China and provided implications for how to improve HPV vaccination coverage among Chinese women of appropriate age, also highlighted the potential role of HPV information exposure in preventive medicine and public health strategies. Ortiz et al. (24) suggested that adolescents are generally interested in receiving information about HPV and the vaccine through social media channels, as long as their privacy is protected and the source is considered credible. It has also been demonstrated that HPV awareness can be increased through brief participation in an online social media platform and receipt of tailored health messages (23). Therefore, public health communication should be further encouraged to deliver HPV and vaccine related information to target groups by taking advantage of the social media, as one of the intervention routes to improve HPV vaccine awareness and further expand HPV vaccine coverage in China. These two findings could further be examined in other studies related to the effect of information exposure on intention to receive other vaccines in China to expand their applicability.

### 5.2. The relationships between knowledge about HPV, attitude toward HPV vaccine and intention to receive the HPV vaccine

We developed a structural equation model using KAP theory and then explored the influence paths of HPV related information exposure, namely, whether increased knowledge about HPV after exposure to HPV information would influence attitude toward HPV vaccine and intention to receive HPV vaccine. KAP theory divided the generation process of individual behavior into three stages: knowledge accumulation (K), attitude formation (A), and behavior promotion (*P*). According to the theory, there is a positive and progressive relationship between knowledge, attitude and practice, that is, knowledge enhances attitude and attitude promotes practice. Our study results suggested that knowledge about HPV was positively related to attitude toward HPV vaccine, and that attitude toward HPV vaccine positively affected the intention to receive HPV vaccine. This result is in accordance with the basic logic of KAP theory and also ties well with other previous vaccine studies using this theory. For example, a KAP survey about pediatric vaccine among pregnant women in the city of Rome found that a good level of knowledge was the strongest predictor of positive attitude toward vaccination, which in turn influenced the majority of respondents' intentions to vaccinate (64).

Surprisingly, a negative association between knowledge about HPV and intention to receive HPV vaccine was found in the results, which was contrary to prior studies (36). Firstly, some authors have speculated that this may be related to individual knowledge about relevant side-effects or adverse reactions of vaccines. In a research on HPV knowledge, behavior and attitude among nursing students in Turkey, Bal-Yilmaz and Koniak-Griffin (65) found a high level of HPV knowledge, however, this was accompanied by a low level of HPV vaccine acceptance at a rate of 14.4%, with a lack of confidence in vaccine efficacy and safety concerns as the most common causes of vaccine refusal. This result was also supported by several studies on parents' intentions to vaccinate their children against HPV (66–68). One of the most frequent reasons for vaccine hesitancy reported to WHO by countries around the world during 2014–2016 was the issue of vaccine safety (69). It is clear that the safety issue has always been a very significant factor affecting vaccination. With the increase in reports of adverse vaccine events in recent years, the level of public trust in vaccine safety has been impacted time and again. For instance, after the Changsheng vaccine incident in 2018, the public in China became skeptical about the vaccine safety and the reliability of vaccine suppliers, especially domestic vaccine manufacturers, and public confidence in the vaccination management system once dropped to a historically low level (70). The “Hong Kong gray-market HPV vaccines” incident in May 2019 has once again caused public doubts about HPV vaccine safety (71), which will seriously hamper the public vaccination in the future. Therefore, the relevant authorities need to consider the public's concerns about the vaccine by not only carrying out studies to test the safety and efficacy of vaccine, but also improving transparency on issues such as vaccine-related injuries and adverse reactions (72), thereby enhancing public trust in vaccine safety and reducing HPV vaccine hesitation.

Secondly, we also speculated that this result might be explained by the perceived cost to the individual, with studies demonstrating that the high price of vaccines was a major cause of vaccine hesitancy, and that even if people were better informed about vaccines, the perceived high cost in terms of time, price, and availability would deter their vaccination (73). However, at this stage, there are still few pilot cities in China where girls of appropriate age can receive the domestic bivalent HPV vaccine for free, and the age is basically limited to women under 18 years old, so most women still need to pay for the HPV vaccine themselves. The domestic supply of HPV vaccine still relies on imports, and the price of imported vaccine is around 1,800–3,900 Yuan (253–548 USD), while the per capita disposable income in China in 2022 is only 3,072 Yuan (432 USD) according to the National Bureau of Statistics (74).^2^ The cost of vaccination may be a greater financial burden for some women of appropriate age, which may become one of the barriers to their HPV vaccination. Our speculation was also proven in some studies related to HPV vaccination in developing countries, a study by Joshi et al. (75) reported that high cost of the vaccine and unavailability of proper and convincing information about the vaccine would be the chief reasons for poor acceptability of HPV vaccine. Therefore, relevant authorities should further facilitate the free HPV vaccination policy to achieve larger population coverage, and also increase multi-sectoral collaboration to address the shortage of HPV vaccine supply and better meet the needs of vaccination.

### 5.3. The effect of injunctive norms on attitude toward HPV vaccine and intention to receive the HPV vaccine

It is notable that our study found that injunctive norms positively affected the attitude toward HPV vaccine and the intention to receive HPV vaccine. This finding is similar to previous studies from other countries, such as a cross-sectional study of HPV vaccination among US college students, which noted that US college students' perception of being expected by their parents and health care providers to get the HPV vaccine influenced their attitudes toward HPV vaccine and further influenced their intentions to get the vaccine (76). In past studies on social norms, scholars have mostly focused on exploring the effects of descriptive norms on individual behaviors, while injunctive norms have received less attention. Through a review of previous literature, Chung and Rimal argued that the two kinds of social norms were more influential in different situations, respectively, depending on whether the behaviors people engage in were positive or negative. Specifically, descriptive norms are more effective in encouraging prosocial behaviors (e.g., health promotion, energy conservation and environmental protection), whereas injunctive norms are more effective in inhibiting various undesirable behaviors (e.g., smoking, alcohol abuse, drug use) (77). However, the results of this study demonstrated the significant effectiveness of injunctive norms in changing young women's attitudes toward HPV vaccine and promoting HPV vaccination as a positive behavior. This may explain the phenomenon of “vaccine fever” in the context of China's unique collectivistic culture. The deep-rooted Confucianism in Chinese culture emphasizes the collectivistic values of “family and nation as one” and “group before individual,” resulting in a strong collectivistic atmosphere in China (78). In this cultural context, the intention to receive HPV vaccine comes not only from self-orientation but also from social orientation, and the vaccination reflects the responsibility and obligation under the social expectation to a greater extent.

However, behind the “vaccine fever,” we should see more clearly that some domestic women of the right age are blind and irrational about HPV vaccination, pursuing the quadrivalent and nine-valent vaccines, and in turn missing the best vaccination period as a result of waiting for quadrivalent and nine-valent vaccines that are currently in short supply. The WHO recommended that the best time for HPV vaccination was between the ages of 9 and 14 years, and therefore, HPV vaccination should be given as early as possible to achieve better effects. Accordingly, in the subsequent promotion of HPV vaccine, a four-level linkage of “government-healthcare sector-community-family” should be applied to health education. It is important to encourage women of the appropriate age to actively receive HPV vaccine under the role of social culture and social norms, as well as to improve HPV-related knowledge among them to reduce irrational vaccination.

Additionally, peer education is one of the key tools to increase vaccine awareness and promote vaccination. Peer-led education programming is currently a prevailing strategy for adolescent sexual and reproductive health education (79), and a series of established studies have demonstrated that using peer education and training not only helps to increase awareness and prevention of HPV and cervical cancer among youth (80), but also has a significant effect on facilitating HPV vaccination (81, 82). Mellanby et al. (83) also found that peer influence is strong within adolescent relationships, and peers may be seen as a more credible source than adult health educators. Also, Chinese women are relatively conservative about sex-related topics, and using the accessibility of peer education can make up for young women's shyness about sex-related topics in adult health education. Selecting appropriate groups for training on HPV knowledge, and then having them disseminate and educate among their peers, can play a supplementary role in the promotion of HPV vaccine to a certain extent.

## 6. Conclusions

Under the context of the HPV vaccination craze, this study was designed to investigate the effects of HPV related information exposure and injunctive norms on young Chinese women's intention to receive the HPV vaccine. On the basis of KAP theory, we proposed a structural equation model containing the influence paths of information exposure and injunctive norms. Through a questionnaire survey and the model testing, we found that exposure to HPV related information had an impact not only on young women's intention to receive HPV vaccine, but also on their knowledge level about HPV. The more frequently they were exposed to HPV related information, the stronger their intentions to get vaccinated and the higher their level of relevant knowledge. Also, the perception and approval of HPV vaccination by those around them further influenced their attitudes and intentions to receive the vaccine. Furthermore, this study confirmed the relationship between knowledge, attitudes, and practice regarding HPV vaccination, with results indicating that good possession of HPV knowledge led to more positive attitudes toward HPV vaccine, and in turn strengthened the intention to receive HPV vaccine. However, a negative association between knowledge level of HPV and intention to be vaccinated was also unexpectedly found, and we speculated that this result might be related to public concerns about vaccine safety issues and the high price of HPV vaccine in China. The findings of this study are of important practical implications for the public health departments and health information service providers to carry out HPV related health education and propel HPV vaccination policies.

## 7. Limitations and future work

Despite the theoretical values and practical significance of this study, there are several limitations that need further improvement in the future research. First, this study was analyzed based on the cross-sectional data obtained from a questionnaire survey, and the study can be deepened in the future by combining qualitative interviews and other methods to explain the individual differences and enhance the dependability of causal inferences. Second, the items measuring information exposure contained both active and passive exposure, and different kinds of information exposure may have different effects on HPV vaccination. Therefore, future research should be undertaken to subdivide the variable of information exposure and explore the different effects of active and passive information exposure. Third, the positive association between HPV knowledge and intention to receive the vaccine was not supported in this study, and factors such as exposure to negative vaccine information and perceived cost could be considered for inclusion in future studies and explored for their moderating effects on the relationship between HPV knowledge and intention to receive the vaccine.

## Data availability statement

The raw data supporting the conclusions of this article will be made available by the authors, without undue reservation.

## Ethics statement

The studies involving human participants were reviewed and approved by Academic Committee of School of Journalism and Communication, Huaqiao University. Written informed consent from the participants' legal guardian/next of kin was not required to participate in this study in accordance with the national legislation and the institutional requirements.

## Author contributions

YW and YC contributed to conception and design of the study. YC wrote the first draft of the manuscript, designed the model and questionnaires, and performed the data analysis. YW and SB collected the data and revised and edited the manuscript. All authors contributed to the article and approved the submitted version.

## Footnotes

^1^ http://gdspma.org.cn/sep-info/849.html

^2^ https://data.stats.gov.cn/english/easyquery.htm?cn=B01
